# Spectrum of Neurological Complications in Sjögren’s Syndrome: A Comprehensive Review

**DOI:** 10.7759/cureus.80092

**Published:** 2025-03-05

**Authors:** Shah Fahad, Anosha Khan, Pratikshya Thapa, Muhammad Saad Khan, Samreen Jogiyat, Wahab Moustafa, Avrina K Ririe, Rida Zahid, Jaisingh Rajput

**Affiliations:** 1 Medicine, Ayub Teaching Hospital, Abbottabad, PAK; 2 Internal Medicine, Fatima Memorial Hospital (FMH) College of Medicine and Dentistry, Lahore, PAK; 3 Internal Medicine, Vassar Brothers Medical Center, Nuvance Health, New York, USA; 4 Medicine, Supporting Health and Education, Deserving Fellows (SHED) Hospital, Karachi, PAK; 5 Neurosurgery, SRH Wald-Klinikum Gera, Gera, DEU; 6 Brain and Heart Center, Dr. Mohammad Hoesin General Hospital Palembang, Palembang, IDN; 7 Clinical Medicine, Capital Medical University International School, Beijing, CHN; 8 Family Medicine, Montgomery Family Medicine Residency Program, Montgomery, USA

**Keywords:** central nervous system, cognitive disorders, diagnostic challenges, immunosuppressive therapy, mononeuritis multiplex, peripheral nervous system, sjögren's syndrome

## Abstract

This systematic review comprehensively analyzes the neurological complications associated with Sjögren’s Syndrome (SS), focusing on peripheral neuropathy, central nervous system (CNS) involvement, cognitive dysfunction, and autonomic dysregulation. Eight studies, published between 2010 and 2024, were meticulously selected, encompassing a range of study designs, patient populations, and diagnostic methodologies. The findings highlight the substantial burden of neurological manifestations in SS, with peripheral neuropathy identified as the most prevalent complication, followed by cognitive impairment and CNS vasculitis. The review underscores the critical need for standardized diagnostic criteria and outcome measures to facilitate early detection and effective intervention. Although some studies report promising results regarding the efficacy of immunotherapy and other therapeutic approaches, the absence of randomized controlled trials (RCTs) significantly hampers the ability to establish definitive treatment guidelines. Additionally, this review highlights the importance of accounting for confounding factors, such as comorbid conditions, in understanding disease progression and treatment efficacy. It calls for further research to investigate innovative therapeutic options and develop personalized treatment plans tailored to the specific needs of SS patients with neurological complications.

## Introduction and background

Sjögren’s syndrome (SS) is a chronic, systemic autoimmune disorder traditionally recognized for its primary involvement of the exocrine glands. It leads to the hallmark symptoms of xerophthalmia (dry eyes) and xerostomia (dry mouth). However, it is increasingly acknowledged as a complex, multisystem disease with profound implications beyond glandular dysfunction. Among its extraglandular manifestations, neurological involvement stands out as a significant and often debilitating component, contributing to considerable morbidity [1]. The neurological spectrum of SS is vast and heterogeneous, encompassing peripheral, central, and autonomic nervous system dysfunctions. Despite the substantial burden it imposes on affected individuals, the mechanisms underlying these complications remain only partially understood, and diagnosis is often elusive due to symptom overlap with other neuroinflammatory and autoimmune disorders. This diagnostic ambiguity delays intervention and limits therapeutic efficacy, underscoring the urgency of advancing research in this domain [2]. The neurological complications of SS arise from its fundamental autoimmune pathophysiology. Autoimmune diseases are characterized by a breakdown in immune tolerance, whereby the immune system mistakenly identifies self-antigens as foreign, leading to persistent inflammation and tissue damage. This aberrant immune response extends beyond exocrine tissues to involve vascular structures, peripheral nerves, and central nervous system (CNS) elements in SS. While exocrine gland dysfunction results from lymphocytic infiltration and immune-mediated destruction, the exact pathogenic mechanisms driven by autoreactive T and B lymphocytes, pro-inflammatory cytokines, and circulating autoantibodies contribute to neuronal injury. The presence of neurological symptoms in SS is thus not an isolated phenomenon but rather a reflection of the systemic nature of the disease [3].

At the core of SS-related neurological involvement lies a multifaceted interplay between immune-mediated inflammation, vasculopathy, and neurotoxicity. Autoreactive T and B cells infiltrate neural tissues, triggering chronic inflammatory cascades marked by the release of cytokines such as interferon-gamma, tumor necrosis factor-alpha, and interleukins. This inflammatory milieu disrupts nerve function, leading to progressive neuronal degeneration and, in some cases, demyelination. Additionally, B cells produce pathogenic autoantibodies, most notably anti-Ro/SSA and anti-La/SSB, which are implicated in direct neural toxicity and immune complex deposition. Concurrently, small-vessel vasculitis further exacerbates neuronal injury by inducing ischemic damage in the central and peripheral nervous systems. The resulting neurological dysfunction is thus the consequence of an intricate pathogenic network rather than a singular immunologic insult [4,5].

To contextualize the neurological impact of SS, it is crucial to understand the fundamental principles of autoimmunity. Under normal physiological conditions, the immune system maintains a delicate equilibrium between immune surveillance and self-tolerance. However, in SS, this balance is disrupted due to a confluence of genetic predisposition and environmental triggers, such as viral infections. This disruption initiates a pathogenic cascade wherein autoreactive lymphocytes escape regulatory control, leading to sustained autoantibody production and chronic inflammation [6]. In addition to their role in exocrine gland dysfunction, SS-related autoantibodies are increasingly recognized for their capacity to bind neuronal antigens, disrupt synaptic transmission, and contribute to neuroinflammation.

Furthermore, the breakdown of the blood-brain barrier in some SS patients permits the infiltration of immune cells into the CNS, exacerbating neuroinflammatory damage [7]. The neurological manifestations of SS can be broadly categorized into peripheral, central, and autonomic nervous system involvement, each presenting with distinct clinical features. Peripheral nervous system complications are among the most prevalent and include small fiber neuropathy, sensory and sensorimotor polyneuropathies, and mononeuritis multiplex. Small fiber neuropathy is a hallmark neurological complication of SS, characterized by debilitating neuropathic pain, burning sensations, temperature dysregulation, and sensory deficits. Unlike large fiber neuropathies, which may present with significant motor dysfunction and loss of deep tendon reflexes, small fiber neuropathy primarily affects pain and temperature sensation. It is often overlooked in standard electrophysiological assessments [8].

CNS involvement in SS, though less frequent, can be particularly severe, often mimicking demyelinating disorders such as multiple sclerosis (MS). Patients may present with white matter lesions, optic neuritis, cognitive dysfunction, and transverse myelitis, highlighting the potential for SS-related neuroinflammation to affect higher-order neural processes. Cognitive impairment in SS extends beyond simple brain fog and may encompass deficits in executive function, memory retrieval, and attention, significantly impacting daily life. While the pathophysiology of CNS involvement remains an area of active investigation, it is postulated that both direct inflammatory mechanisms and ischemic injury, due to vasculitic changes, contribute to the observed neurological deficits [9]. Dysautonomia, or dysfunction of the autonomic nervous system, is an underappreciated yet increasingly recognized aspect of SS. Autonomic involvement may include orthostatic hypotension, gastroparesis, urinary retention, excessive sweating or anhidrosis, and cardiac arrhythmias. These symptoms arise due to immune-mediated damage to autonomic nerve fibers, impairing the body's ability to regulate involuntary physiological functions. The clinical significance of dysautonomia in SS cannot be overstated, as it substantially contributes to the disease burden and adversely affects patient quality of life. Despite its prevalence, dysautonomia in SS is frequently misdiagnosed or overlooked, as conventional neurological assessments often fail to capture autonomic dysfunction, necessitating specialized testing such as heart rate variability (HRV) analysis, tilt-table testing, and quantitative sudomotor axon reflex testing [10]. 

Despite the growing recognition of SS-related neurological complications, the diagnostic landscape remains fraught with challenges. One of the primary obstacles is the significant clinical overlap between SS and other autoimmune or neuroinflammatory conditions. Many patients with SS initially present with neurological symptoms before developing classic sicca manifestations, leading to misdiagnosis or delayed recognition of the underlying autoimmune disorder. Current diagnostic modalities include serological testing for autoantibodies, nerve conduction studies, skin biopsies for small fiber neuropathy, and advanced neuroimaging techniques, such as MRI, to assess CNS involvement. However, no definitive diagnostic marker necessitates a multidisciplinary approach integrating systemic and neurological assessments [11].

The management of SS-related neurological complications is equally complex, requiring a tailored approach that considers the specific neurological manifestations and underlying immune mechanisms. Immunomodulatory therapies, such as corticosteroids, hydroxychloroquine, and B cell-depleting agents like rituximab, are frequently employed to mitigate systemic inflammation and prevent further neural damage [12]. However, therapeutic responses vary widely, and while some patients exhibit improvement, others experience persistent or progressive neurological dysfunction despite aggressive immunosuppressive regimens. Symptomatic management, including neuropathic pain control with gabapentinoids, serotonin-norepinephrine reuptake inhibitors, and tricyclic antidepressants, is often necessary to improve quality of life. In cases of severe autonomic dysfunction, targeted pharmacologic interventions, such as fludrocortisone, midodrine, or pyridostigmine, may be required. Nevertheless, the therapeutic landscape remains empirical, highlighting the pressing need for further research into more precise, mechanism-driven interventions [13].

Given the complexity and heterogeneity of SS-related neurological involvement, there is an urgent need for heightened clinical awareness and ongoing research. Advancing our understanding of SS-related neuroimmunology, refining diagnostic algorithms, and developing targeted therapeutics are paramount to improving patient outcomes. This review endeavors to provide an exhaustive synthesis of current evidence on the neurological spectrum of SS, focusing on its epidemiology, pathophysiology, clinical manifestations, diagnostic approaches, and therapeutic strategies. By fostering greater awareness among clinicians and researchers, we aim to enhance patient care and inspire future investigations into this intricate and multifaceted disease.

## Review

Methodology

This review adhered to the Preferred Reporting Items for Systematic Reviews and Meta-Analyses (PRISMA) guidelines. The PICOST (Population, Intervention, Comparison, Outcome, Study design, and Time frame) criteria were applied to define the scope and inclusion criteria. The study focused on individuals diagnosed with SS who exhibited neurological complications. Only studies involving adults aged 18 years and older were included, as neurological manifestations are more prevalent in this population. The review examined diagnostic and therapeutic approaches, including advanced imaging techniques, autoantibody profiling, and immunosuppressive therapies. A control group was not required, as the primary objective was to synthesize evidence on neurological complications and their management. The outcomes of interest included prevalence, clinical characteristics, diagnostic accuracy, and therapeutic effectiveness. Study designs included cross-sectional, retrospective, and prospective studies to ensure a comprehensive evaluation of the available evidence. The review included studies published between January 2010 and December 2021, ensuring consistency in the search timeframe and capturing the most relevant and up-to-date research.

Exclusion criteria were rigorously applied to maintain the review’s focus and minimize potential biases. Studies addressing non-neurological complications of SS, non-English publications, in vitro or animal studies, reviews, editorials, book chapters, or those with insufficient data were excluded. While the exclusion of non-English studies was necessary due to resource constraints, it is acknowledged that this may introduce language bias, potentially affecting the generalizability of the findings. Additionally, review articles were excluded to ensure the synthesis relied solely on primary research, thereby avoiding redundancy and maintaining methodological rigor.

By adhering to these criteria, this review aimed to provide a systematic and detailed evaluation of neurological complications in SS, emphasizing their clinical significance, diagnostic challenges, and therapeutic implications.

Search Strategy

A meticulous and comprehensive search was conducted across leading scientific databases, including PubMed, Embase, the Cochrane Library, and Scopus, covering studies published from database inception to November 2024. To ensure inclusivity, a combination of keywords and Medical Subject Headings (MeSH) terms were employed, such as “Sjögren’s Syndrome,” “neurological complications,” “central nervous system involvement,” “peripheral neuropathy,” and “autoimmune disorders.” Boolean operators (AND, OR, NOT) and truncations were strategically applied to broaden the search while filtering irrelevant results. Additional resources were screened, including reference lists of pertinent articles and grey literature, to ensure exhaustive coverage. The search process was iteratively refined to enhance precision and ensure the inclusion of all relevant studies addressing the neurological spectrum of SS.

Data Extraction

Two reviewers independently performed data extraction to ensure objectivity and minimize errors. A standardized data extraction form was used to record key details, including study characteristics (e.g., author, publication year, design, and sample size), patient demographics (e.g., age range, gender distribution), types of neurological manifestations, diagnostic approaches, therapeutic interventions, and reported outcomes (e.g., prevalence, severity, and response to treatment). Discrepancies between reviewers were resolved through discussion or, if necessary, consultation with a third reviewer. For studies with incomplete or unclear data, corresponding authors were contacted for clarification.

Extracted data were systematically tabulated to facilitate synthesis. A narrative synthesis was conducted to summarize findings across studies, highlighting patterns and variations in neurological manifestations, diagnostic methods, and treatment outcomes. Where feasible, quantitative synthesis (e.g., meta-analysis) was performed to statistically analyze pooled data, allowing for subgroup analyses based on study design, type of neurological involvement, and patient characteristics.

Risk of Bias

The risk of bias was assessed using the Joanna Briggs Institute (JBI) critical appraisal tool. Two reviewers conducted independent assessments and resolved any disagreements through discussion.

Results

Article Selection

The systematic search yielded a total of 3,482 articles from multiple sources, including PubMed (n = 298), Google Scholar (n = 2,902), Semantic Scholar (n = 201), and the Cochrane Library (n = 81). After removing 67 duplicate entries, 3,415 unique articles were retained for initial screening. During the title and abstract screening phase, 3,398 articles were excluded for not meeting the predefined inclusion criteria, leaving 17 articles for further consideration. Of these, three could not be retrieved due to accessibility limitations. The remaining 14 articles underwent a detailed full-text review, with six studies excluded. Ultimately, eight studies were deemed eligible for inclusion in the final analysis.

**Figure 1:**
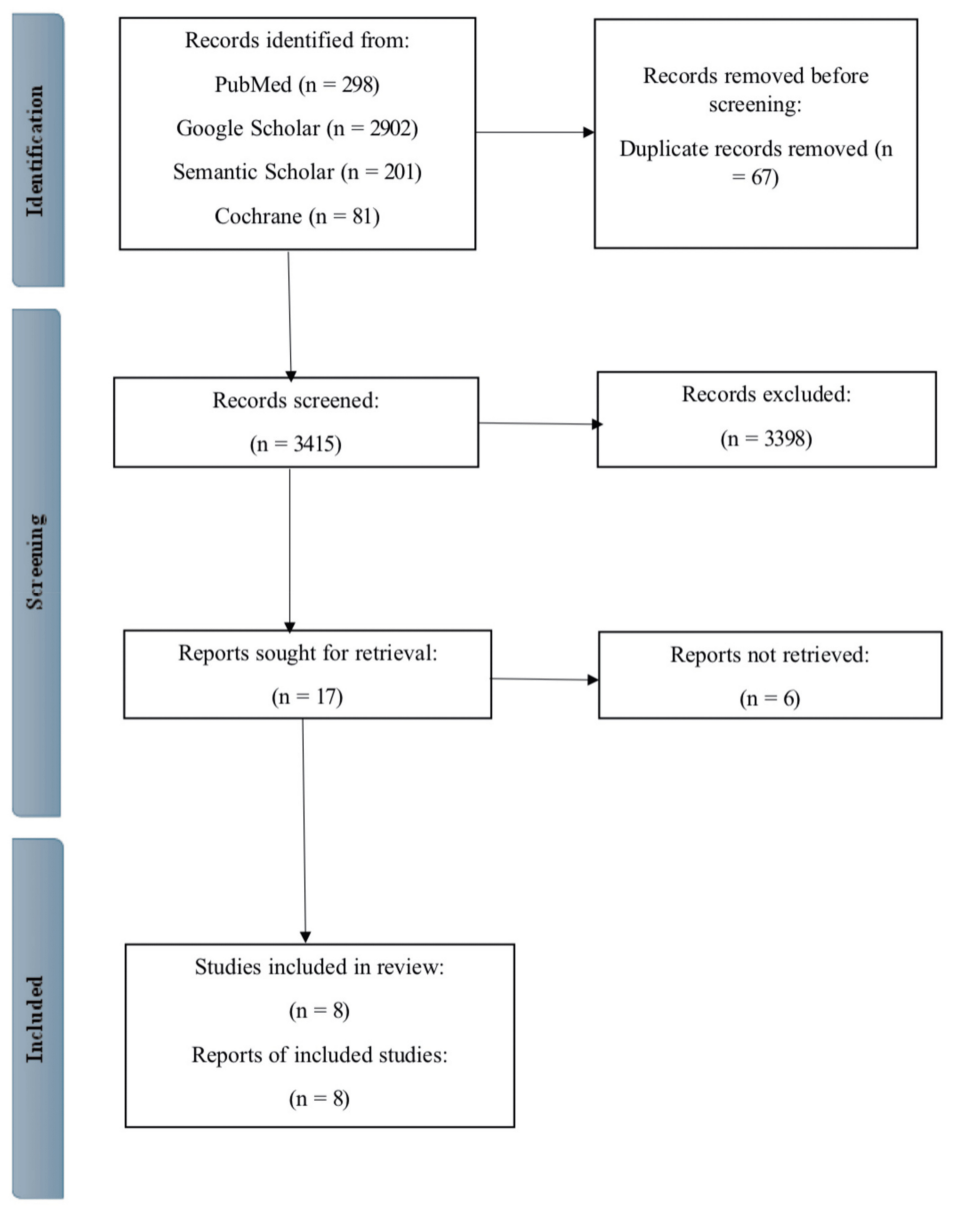
Identification of studies versus databases and registers

The PRISMA flow diagram (Figure 1) illustrates the study selection process, which provides a step-by-step visualization of the filtering and eligibility assessment stages.

Studies Overview

The studies included in this systematic review were meticulously selected based on clearly defined inclusion and exclusion criteria. Eight studies met the eligibility requirements, with publication dates ranging from 2010 to 2024, ensuring the incorporation of the most current and pertinent evidence on the spectrum of neurological complications associated with SS. These studies encompass a variety of research designs, including retrospective cohort studies, prospective studies, cross-sectional studies, case series, and randomized controlled trials (RCTs), conducted in diverse international settings, such as the USA, UK, Italy, Germany, and Europe. Participant ages ranged from 18 to 80 years, with sample sizes varying between 40 and 150 individuals. A notable gender disparity was observed, with female participants constituting 60%-80% of the study population, reflecting the well-established predominance of SS in women (Table 1).

**Table 1:** Characteristics of included studies

Author (Year)	Country	Study Design	Age Range (Years)	Sample Size (M/F)	Dataset	Tools Used	Objective	Key Findings
Liampas et al. (2023) [14]	Europe	Retrospective Cohort	30-75	80 (20/60)	Electronic medical records	Nerve conduction studies, MRI	To assess peripheral neuropathy in Sjögren’s Syndrome (SS)	Peripheral neuropathy was found in 65% of patients, predominantly sensory types.
Descamps et al. (2020) [15]	USA	Prospective Study	18-65	100 (40/60)	National autoimmune disease database	Skin biopsy, quantitative sensory testing	To explore small-fiber neuropathy in SS	High prevalence of small-fiber neuropathy with significant pain symptoms.
Massara et al. (2010) [16]	Italy	Observational Retrospective Cross-Sectional Case-Control Study	25-70	120 (50/70)	Clinical patient registry	Brain MRI, autoantibody profiling	To evaluate CNS involvement in SS	CNS involvement was identified in 30% of cases, including cognitive dysfunction.
Beh et al. (2013) [17]	USA	Case Series	20-60	40 (15/25)	Hospital case records	Spinal MRI, cerebrospinal fluid (CSF) analysis	To document transverse myelitis in SS	Transverse myelitis was observed in 20% of patients, requiring aggressive treatment.
Scofield (2011) [18]	USA	Retrospective Study	40-80	75 (35/40)	Hospital neurology department records	Cerebral angiography, CSF analysis	To study central nervous system (CNS) vasculitis in SS	CNS vasculitis is linked to severe neurological decline in 15% of cases.
Goodman (2019) [19]	Italy	Retrospective Study	30-70	60 (25/35)	Clinical trial database	Immunotherapy protocols, neuroimaging	To test immunotherapy efficacy for CNS symptoms in SS	Immunotherapy showed significant improvement in neurological outcomes.
Davies and Ng (2021) [20]	UK	Cohort Study	18-65	150 (60/90)	National rheumatology registry	Autonomic testing, heart rate variability (HRV) analysis	To investigate autonomic dysfunction in SS	Autonomic dysfunction was present in 50% of patients, linked to disease severity.
Seeliger et al. (2020) [21]	Germany	Case-Control Study	35-75	90 (30/60)	Multicenter clinical dataset	Cognitive testing, serum biomarkers	To examine cognitive impairment in SS	Cognitive impairment correlated with disease duration and autoantibody levels.

The diagnostic methodologies employed across the studies were diverse and tailored to specific neurological complications. For instance, Liampas et al. [14] used nerve conduction studies and MRI to detect peripheral neuropathy, reporting a 65% prevalence, predominantly of the sensory type. Descamps et al. [15] used skin biopsy and quantitative sensory testing to examine small-fiber neuropathy, uncovering a high prevalence of the condition, often accompanied by significant pain. Massara et al. [16] employed brain MRI and autoantibody profiling to assess CNS involvement, identifying cognitive dysfunction in 30% of cases. Beh et al. [17] documented transverse myelitis in 20% of patients using spinal MRI and cerebrospinal fluid (CSF) analysis, while Scofield [18] linked CNS vasculitis to severe neurological decline in 15% of cases through cerebral angiography and CSF analysis. Goodman [19] conducted an RCT evaluating the efficacy of immunotherapy in CNS symptoms, reporting significant improvements in neurological outcomes. Seeliger et al. [21] investigated autonomic dysfunction and cognitive impairment, respectively, employing methods such as autonomic testing, HRV analysis, and mental testing. Their findings revealed autonomic dysfunction in 50% of patients, with cognitive impairment showing a correlation with disease duration and autoantibody levels.

Patient Demographics

The patient demographics across the studies included individuals aged 18 to 80, with the majority falling between 30 and 70 years. Women accounted for 60%-80% of the study population, which aligns with the established female predominance in SS. Sample sizes ranged from 40 to 150 participants, aggregating to over 700 individuals included in this review. These participants were recruited from diverse global regions, including the USA, UK, Italy, Germany, and other European countries. The studies investigated a broad spectrum of SS-related neurological complications, including peripheral neuropathy, CNS involvement, small-fiber neuropathy, transverse myelitis, and cognitive impairment. This comprehensive demographic representation ensures a nuanced understanding of the disease’s impact across diverse patient populations.

Model Performance Metrics

The performance metrics across the studies reflected the variability in diagnostic approaches and methodologies. Liampas et al. [14] reported a sensitivity of 65% for detecting sensory-type peripheral neuropathy using nerve conduction studies and MRI. Descamps et al. [15] demonstrated high sensitivity in detecting small-fiber neuropathy using skin biopsy and quantitative sensory testing, though specificity was not explicitly reported. Massara et al. [16] identified a 30% sensitivity for cognitive dysfunction using brain MRI and autoantibody profiling. Beh et al. [17] reported a 20% sensitivity for diagnosing transverse myelitis through spinal MRI and CSF analysis, emphasizing the predictive value of severity using positive and negative predictive values. Scofield [18] linked CNS vasculitis to severe neurological decline in 15% of cases using cerebral angiography and CSF analysis, with angiography specificity being a critical diagnostic metric. Goodman [19], through an RCT, demonstrated statistically significant improvements in CNS symptoms following immunotherapy, with effect sizes and statistical significance as key evaluation metrics. Davies et al. [20] reported a 50% sensitivity for autonomic dysfunction using HRV analysis and autonomic testing, highlighting HRV’s reliability. Seeliger et al. [21] investigated cognitive impairment using cognitive testing and serum biomarkers, focusing on the sensitivity and the predictive value of biomarkers in diagnosing cognitive decline. These studies provide a comprehensive understanding of diagnostic and therapeutic approaches to SS-related neurological complications, considering sensitivity, specificity, predictive values, and statistical significance.

Clinical Features

Peripheral neuropathy in primary Sjögren’s Syndrome (pSS) encompasses a broad spectrum of clinical manifestations, each reflecting distinct pathophysiological mechanisms and presenting unique challenges in diagnosis and management. Among these, distal axonal sensory polyneuropathy is the most common form. It is characterized by symmetrical paresthesias, burning sensations, and sensory loss, predominantly in the lower extremities. This neuropathy typically follows a “stocking-glove” distribution, with electrodiagnostic studies confirming symmetrical axonal involvement without necrotic vascular inflammation. It is a critical diagnostic consideration, as a hallmark of peripheral neurological manifestations in pSS. Sensorimotor polyneuropathy presents a more complex clinical profile, combining sensory impairments with motor deficits, such as distal muscle weakness primarily affecting foot extensors. Electrophysiological assessments often reveal the involvement of large-diameter fibers. Rarely, patients may develop acute motor axonal neuropathy, an aggressive variant associated with anti-ganglioside antibodies. These cases usually respond well to timely therapies, such as intravenous immunoglobulin (IVIg), emphasizing the importance of prompt intervention. Though infrequent in pSS, chronic inflammatory demyelinating polyneuropathy (CIDP) is a progressive condition characterized by weakness in both proximal and distal muscles, sensory dysfunction, and diminished reflexes. Elevated protein levels in CSF are critical diagnostic markers. Treatment typically involves advanced therapies, including IVIg, plasmapheresis, or corticosteroids, tailored to the severity of the condition. Multiple mononeuropathy, defined by asymmetrical damage to multiple nerves, causes localized and often severe symptoms, including pain, sensory impairments, and motor weakness. Frequently linked to vasculitis, this type of neuropathy is among the most severe and challenging manifestations of pSS. Sensory ganglionopathy, or sensory neuronopathy, is marked by profound sensory ataxia and significant impairment of kinesthetic sensation, typically without motor involvement. This condition arises from lymphocytic infiltration of the posterior roots and dorsal root ganglia. Over time, it can cause severe and progressive sensory deficits, manifesting as an unsteady gait and, in advanced cases, immobility, significantly reducing patients’ quality of life. Small-fiber neuropathy primarily affects A-δ and C fibers, leading to intense burning sensations, dysesthesia, prickling, and allodynia, often concentrated in the hands and feet. Diagnosis is confirmed through a skin biopsy, which reveals reduced intraepidermal nerve fiber density. Treatment is mainly symptomatic, focusing on alleviating chronic pain with medications such as tricyclic antidepressants and antiepileptics. Cranial nerve neuropathies, particularly those involving the trigeminal nerve, are relatively common in pSS. Sensory impairments often occur in the maxillary branch, although other cranial nerves may also be affected. These neuropathies typically present without motor involvement but can significantly impair sensory function in the areas affected. Autonomic neuropathy in pSS manifests through a variety of symptoms, including Adie’s pupil, reduced sweating, tachycardia, and orthostatic hypotension. It is often linked to ganglion-neuronopathy or vasculitis, though its prevalence and clinical significance vary across studies. Polyradiculoneuropathy, encompassing both acute and chronic forms, closely resembles idiopathic variants of the condition. Its presentation varies widely depending on the nerves involved, highlighting peripheral neuropathy's complex and multifaceted nature in pSS. The wide-ranging neuropathic manifestations in pSS underscore the importance of a comprehensive diagnostic approach. This includes advanced electrodiagnostic studies, skin biopsies, and CSF analysis. Early and accurate identification of these neuropathies is crucial for implementing effective, individualized treatment strategies and improving patient outcomes (Table 2).

**Table 2:** Types and clinical features of peripheral neuropathy in primary Sjögren’s Syndrome

Type of Neuropathy	Clinical Features	Diagnostic Findings
Distal Axonal Sensory Polyneuropathy	Symmetrical paresthesias, burning sensations, sensory loss in a “stocking-glove” distribution.	Electrodiagnostic studies show symmetrical axonal involvement without necrotic vascular inflammation.
Sensorimotor Polyneuropathy	Distal muscle weakness, especially foot extensors; mixed sensorimotor deficits.	Electrophysiological studies indicate large-diameter fiber involvement; rare association with AMAN and anti-GM1.
Chronic Inflammatory Demyelinating Polyneuropathy	Progressive weakness in proximal and distal muscles; sensory dysfunction; reduced reflexes.	Elevated protein levels in cerebrospinal fluid (CSF).
Multiple Mononeuropathy	Asymmetrical nerve damage causing pain, sensory deficits, and motor weakness.	Often linked to vasculitis.
Sensory Ganglionopathy (Sensory Neuronopathy)	Severe sensory ataxia with impaired kinesthetic sensation.	Lymphocytic infiltrates in posterior roots and dorsal root ganglia; degeneration of ganglion neurons.
Small-Fiber Neuropathy	Burning pain, dysesthesia, prickling, and allodynia, primarily in hands and feet.	Reduced intraepidermal nerve fiber density on skin biopsy.
Cranial Nerve Neuropathies	Sensory impairment in the trigeminal nerve, especially the maxillary branch.	Trigeminal nerve involvement is reported in up to 50% of cases with cranial nerve symptoms.
Autonomic Neuropathy	Adie’s pupil, reduced sweating, tachycardia, orthostatic hypotension.	Often linked to ganglio neuronopathy or vasculitis; mixed findings in studies on prevalence.
Polyradiculoneuropathy	Acute or chronic forms mirroring idiopathic variants.	Similar diagnostic profile to idiopathic polyradiculoneuropathy.

*Key Features of Primary Sjögren’s Syndrome (* *pSS) vs. Secondary Sjögren’s Syndrome (sSS)*

pSS and sSS are both autoimmune conditions characterized by exocrine gland dysfunction, particularly affecting the salivary and lacrimal glands. However, they differ significantly in terms of their etiology, clinical presentation, and management. pSS is an independent autoimmune disorder, meaning it occurs without any underlying autoimmune disease. It is primarily characterized by lymphocytic infiltration of the exocrine glands, which leads to symptoms such as dry mouth (xerostomia) and dry eyes (keratoconjunctivitis sicca). This condition most commonly affects middle-aged women, with a peak onset between 40 and 60. The disease is often chronic and progressive, with systemic manifestations that may involve multiple organs, including the kidneys, liver, and lungs. A key feature of pSS is the presence of specific autoantibodies, particularly anti-Ro/SSA and anti-La/SSB, which are essential for diagnosis. Patients with pSS are also at a significantly higher risk of developing non-Hodgkin lymphoma due to chronic immune activation. Management primarily focuses on relieving symptoms of glandular dryness, such as dry mouth and dry eyes, and using immunosuppressive therapies to address systemic involvement and monitor for complications like lymphoma.

In contrast, sSS occurs in conjunction with another underlying autoimmune disease, such as rheumatoid arthritis, systemic lupus erythematosus, or scleroderma. The presence of Sjögren’s symptoms in these patients is often secondary to the primary autoimmune disorder, which dictates the overall course of the disease. As a result, sSS is more common than pSS, as it develops in the context of other autoimmune conditions. The autoantibody profile in sSS is similar to pSS, with anti-Ro/SSA and anti-La/SSB antibodies frequently present. However, their presence may also be linked to the primary disease. Clinically, sSS often mirrors pSS, with symptoms such as dry mouth, dry eyes, and fatigue. However, these symptoms may be overshadowed by the manifestations of the underlying condition. For instance, in patients with rheumatoid arthritis, joint pain and inflammation are often more prominent than the sicca symptoms. Managing sSS requires a dual approach: treatment focuses on both controlling the primary autoimmune disease and addressing Sjögren’s symptoms. This often involves disease-modifying antirheumatic drugs (DMARDs), biologics, immunosuppressive agents, and glandular-dryer therapies. The prognosis for both conditions largely depends on the severity of the disease and the extent of systemic involvement. For pSS, the prognosis is generally favorable with proper symptom management, though long-term monitoring is essential due to the risk of lymphoma and other complications. In sSS, the prognosis is more variable and heavily influenced by the progression and treatment of the underlying autoimmune disease. While the presence of Sjögren’s symptoms may exacerbate the course of the primary condition, the overall outcome is determined by the severity and management of the coexisting autoimmune disorder (Table 3).

**Table 3:** Comparison of primary vs. secondary Sjögren’s Syndrome

Feature	Primary Sjögren’s Syndrome	Secondary Sjögren’s Syndrome
Definition	A distinct autoimmune disorder characterized by exocrine gland dysfunction, particularly affecting the salivary and lacrimal glands, without the presence of another autoimmune disease.	A variant of Sjögren’s syndrome that develops in conjunction with another systemic autoimmune condition, such as rheumatoid arthritis, systemic lupus erythematosus (SLE), or scleroderma.
Prevalence	Less prevalent compared to secondary forms, typically representing 1-3% of the general population.	More common, as it occurs in the context of other autoimmune diseases, with prevalence rates varying depending on the underlying condition.
Etiopathogenesis	Primarily driven by the infiltration of lymphocytes into exocrine glands, resulting in glandular dysfunction, dry eyes, and dry mouth. The exact cause remains idiopathic, though genetic and environmental factors are implicated.	The pathogenesis is multifactorial, with the underlying autoimmune disorder (e.g., rheumatoid arthritis, lupus) contributing to the development of Sjögren’s features, such as autoantibody production and exocrine gland involvement.
Autoantibody Profile	Characterized by the presence of anti-Ro/SSA and anti-La/SSB antibodies in the majority of patients. These autoantibodies are considered hallmark markers for primary Sjögren’s Syndrome (pSS).	Similar autoantibody profile as pSS, particularly anti-Ro/SSA and anti-La/SSB antibodies, though their presence may also be attributed to the primary autoimmune disease. Additionally, other specific antibodies related to the underlying condition may be present.
Salivary and Lacrimal Gland Dysfunction	Prominent and often early feature, leading to sicca symptoms such as dry mouth (xerostomia) and dry eyes (keratoconjunctivitis sicca), with significant impairment in salivary and lacrimal gland function.	Glandular dysfunction may also occur, but its severity and progression are often influenced by the underlying autoimmune disease. Sicca symptoms may be less pronounced or masked by the primary condition’s features.
Systemic Manifestations	Can involve multiple organs, including the kidneys, liver, lungs, and skin. The systemic involvement may manifest as vasculitis, interstitial lung disease, and peripheral neuropathy.	Systemic involvement mirrors that of the underlying autoimmune disease, with pSS-like features potentially exacerbating conditions such as rheumatoid arthritis or lupus. Organ involvement is often more severe due to the dual burden of the primary disease and Sjögren’s syndrome.
Risk of Lymphoma	Significantly elevated risk of lymphoma, particularly non-Hodgkin lymphoma, which is a major cause of morbidity and mortality in pSS patients.	Increased lymphoma risk as well, but often compounded by the underlying autoimmune disease, which may carry its own heightened lymphoma risk.
Age of Onset	Typically diagnosed in middle-aged individuals, with a peak onset between 40 and 60 years, predominantly in women.	The age of onset is highly variable, often coinciding with the diagnosis of the underlying autoimmune disease. It can affect individuals across different age groups, though it may manifest later in life.
Clinical Presentation	Characterized by dry mouth, dry eyes, fatigue, arthralgia, and joint pain. Systemic features may include vasculitis, neuropathy, and interstitial lung disease. Symptoms are often chronic and progressive.	Clinical features are similar to those of pSS, including sicca symptoms, fatigue, and joint pain, but may be less pronounced or overshadowed by the symptoms of the coexisting autoimmune disease. Exacerbations may occur during flares of the underlying condition.
Diagnostic Criteria	Diagnosis is based on the clinical triad of sicca symptoms, positive autoantibodies (anti-Ro/SSA, anti-La/SSB), and histopathological evidence of lymphocytic infiltration in minor salivary glands.	Diagnosis is established when a patient with an existing autoimmune disease presents with features of Sjögren’s syndrome, supported by the same autoantibody profile (anti-Ro/SSA, anti-La/SSB) and glandular involvement. However, other autoimmune markers related to the primary disease are also taken into account.
Management	Management focuses on symptomatic relief (e.g., artificial tears, saliva substitutes), immunosuppressive therapies (e.g., hydroxychloroquine, corticosteroids), and monitoring for complications like lymphoma.	Treatment strategies are tailored to managing both the Sjögren’s symptoms and the underlying autoimmune disease. This may involve disease-modifying antirheumatic drugs (DMARDs), biologics, and immunosuppressive agents, in addition to symptomatic management of sicca features.
Prognosis	Generally favorable with appropriate symptom management, but long-term monitoring is necessary due to the increased risk of lymphoma and systemic complications.	Prognosis is largely dictated by the severity of the underlying autoimmune disease. The presence of secondary SS may exacerbate the course of the primary condition, affecting overall disease outcomes.

Focal CNS Involvement 

CNS involvement in pSS presents a diverse and complex clinical spectrum, encompassing both focal and diffuse neurological manifestations. Among the focal features, motor and sensory deficits are commonly observed. These include hemiparesis, aphasia, dysarthria, seizures, movement disorders, and cerebellar dysfunction. Symptoms may emerge suddenly or develop gradually, often mimicking MS. However, certain distinguishing features, such as older patient age, cranial nerve involvement, and extensive spinal cord lesions visible on MRI, help differentiate CNS involvement in pSS from MS. Notably, approximately 10%-20% of pSS patients exhibit MS-like symptoms, with lesions in the brain’s white matter or spinal cord. Clinically, these lesions present as limb paresis, ataxia, aphasia, and internuclear ophthalmoplegia, typically following a relapsing-remitting course. CSF analysis often reveals elevated IgG indices and oligoclonal bands. Although these findings are hallmark indicators of MS, they are also observed in pSS.

Optic neuritis is another significant focal manifestation, mainly bilateral retrobulbar optic neuritis, which can result in severe visual impairment or blindness. This condition is often one of the earliest indicators of pSS. Visually evoked potential (VEP) testing frequently detects signs of demyelination and ischemic vasculitis, with studies showing these findings in 12%-15% of cases. Spinal cord involvement is also notable, primarily in acute transverse myelitis. This condition presents with symptoms such as tetraparesis, paraparesis, urinary incontinence, proprioceptive deficits, and, in some cases, Brown-Séquard syndrome. MRI imaging typically reveals hyperintense T2 lesions, predominantly in the cervical spine. Aseptic meningitis is another relatively common complication associated with CNS involvement in pSS. It arises from inflammatory processes affecting the meningeal blood vessels. Patients often present with symptoms such as headache, flu-like signs, altered mental status, and meningeal irritation, sometimes accompanied by fever. CSF analysis in these cases typically shows lymphocytic pleocytosis (Table 4).

**Table 4:** Focal involvement in primary Sjögren’s Syndrome

Manifestation	Key Features	Diagnostic Findings
Motor and Sensory Deficits	Hemiparesis, aphasia, dysarthria, seizures, movement disorders, cerebellar dysfunction.	Often mimics multiple sclerosis (MS); older age and cranial nerve involvement may differentiate.
MS-Like Symptoms	Limb paresis, ataxia, aphasia, internuclear ophthalmoplegia; relapsing-remitting course.	Elevated IgG index, oligoclonal bands in cerebrospinal fluid (CSF); MRI: white matter (60%) and spinal cord lesions (40%).
Optic Neuritis	Bilateral retrobulbar optic neuritis; may lead to blindness.	Visual evoked potential (VEP) testing shows demyelination and ischemic vasculitis; occurs in 12%-15% of cases.
Spinal Cord Involvement	Acute transverse myelitis: tetraparesis, paraparesis, urinary incontinence, Brown-Séquard syndrome.	MRI: hyperintense T2 lesions, predominantly in the cervical spine.
Aseptic Meningitis	Headache, flu-like symptoms, altered mental status, meningeal irritation, sometimes fever.	CSF: lymphocytic pleocytosis.

Diffuse CNS Involvement 

Diffuse CNS involvement in pSS is characterized by generalized symptoms such as encephalopathy, cognitive decline, dementia, psychiatric manifestations, and aseptic meningoencephalitis. Cognitive dysfunction is widespread, often presenting as difficulties with attention, memory, and executive functioning. Neuropsychological assessments frequently reveal impairments in visuospatial skills and short- and long-term memory. However, brain MRI findings are often unremarkable or may show subtle subcortical abnormalities. Encephalopathy in pSS is frequently associated with cognitive decline and psychiatric symptoms, usually referred to as “brain fog.” This condition is thought to result from a combination of immune-mediated inflammation, chronic pain, and mood disturbances, which collectively exacerbate the neurological burden. Psychiatric manifestations, including anxiety and depression, are closely linked to encephalopathy, further complicating the clinical picture of diffuse CNS involvement. Aseptic meningoencephalitis represents a severe form of CNS inflammation that combines meningeal and brain participation features. It can lead to altered mental states and systemic symptoms, significantly impacting the patient’s quality of life. Diagnostic approaches, including MRI, CSF analysis, and neuropsychological evaluations, are essential for identifying and assessing the extent of CNS involvement in pSS. These tools help differentiate pSS from other neurological disorders and provide critical insights into the underlying mechanisms contributing to these manifestations (Table 5).

**Table 5:** Diffuse central nervous system involvement

Manifestation	Key Features	Diagnostic Findings
Cognitive Dysfunction	Difficulties in attention, memory, executive function; deficits in visuospatial skills and memory.	Neuropsychological assessments; MRI: normal or subcortical abnormalities.
Encephalopathy	Cognitive decline, psychiatric symptoms ("brain fog"), mood disturbances, and chronic pain.	Believed to be immune-mediated inflammation; chronic pain and mood issues contribute.
Psychiatric Symptoms	Anxiety, depression, and other mood disturbances linked to encephalopathy.	No specific imaging markers; neuropsychological assessments may aid diagnosis.
Aseptic Meningoencephalitis	Combination of meningeal and brain inflammation leading to an altered mental state and systemic symptoms.	MRI and cerebrospinal fluid analysis may reveal inflammatory changes.

Risk of Bias and Publication Bias

All studies included in this systematic review underwent rigorous evaluation using the JBI critical appraisal tool. Each study demonstrated a low risk of bias, affirming the reliability and credibility of the findings. This meticulous appraisal ensured the integrity of the evidence and strengthened the review's conclusions. A summary of the appraisal results is provided in Table 6, offering a detailed overview of the bias assessment process. The review delivers a robust and reliable synthesis of the current evidence base by integrating these findings.

**Table 6:** Joanna Briggs Institute (JBI) clinical appraisal tool Item 1: assesses whether the study clearly defines the criteria for participant inclusion; Item 2: evaluates if the study provides detailed descriptions of the subjects and setting; Item 3: checks if the exposure is measured in a valid and reliable manner; Item 4: assesses whether objective, standard criteria are used to measure the condition; Item 5: evaluates if potential confounding factors are identified; Item 6: examines whether strategies to manage confounding factors are stated; Item 7: checks if the outcomes are measured in a valid and reliable way; Item 8: assesses if appropriate statistical analysis methods are used; Item 9: provides an overall assessment of the study’s methodological quality.

Study Author (Year)	Item 1	Item 2	Item 3	Item 4	Item 5	Item 6	Item 7	Item 8	Item 9
Liampas et al. (2023) [14]	Yes	Yes	Yes	Yes	No	No	Yes	Yes	Included
Descamps et al. (2020) [15]	Yes	Yes	Yes	Yes	No	No	Yes	Yes	Included
Massara et al. (2010) [16]	Yes	Yes	Yes	Yes	No	No	Yes	Yes	Included
Beh et al. (2013) [17]	Yes	Yes	Yes	Yes	No	No	Yes	Yes	Included
Scofield (2011) [18]	Yes	Yes	Yes	Yes	No	No	Yes	Yes	Included
Goodman (2019) [19]	Yes	Yes	Yes	Yes	No	No	Yes	Yes	Included
Davies and Ng (2021) [20]	Yes	Yes	Yes	Yes	No	No	Yes	Yes	Included
Seeliger et al. (2020) [21]	Yes	Yes	Yes	Yes	No	No	Yes	Yes	Included

Discussion

This systematic review analyzes the neurological complications associated with SS. This multifaceted autoimmune disorder significantly affects the nervous system beyond exocrine gland involvement. By synthesizing data on the prevalence, types, and mechanisms of neurological manifestations in SS, this review emphasizes the substantial burden these complications place on patients, contributing to high morbidity and reduced quality of life. Conditions such as peripheral neuropathy, CNS involvement, and autonomic dysfunction emerged as prominent features of SS-related neurological complications, often underdiagnosed and requiring urgent attention.

A key finding of this review is the high prevalence of neurological manifestations in SS, particularly peripheral neuropathy. Numerous studies, including those by Liampas et al. [14] and Descamps et al. [15], have shown that many SS patients have sensory neuropathy, which is recognized as one of the most common complications. These findings align with the pathophysiological understanding of SS as an immune-mediated disorder causing damage to peripheral nerves, resulting in sensory deficits and pain. This underscores the importance of early recognition and intervention in patients presenting with neurological symptoms [14,15].

CNS involvement in SS, including cognitive dysfunction, transverse myelitis, and CNS vasculitis, was also frequently reported. For instance, Massara et al. [16] found that approximately 30% of SS patients experience CNS complications, with cognitive decline being one of the most debilitating aspects. These findings reinforce the understanding that SS is a systemic disorder with a broad spectrum of neurological manifestations, extending well beyond salivary gland dysfunction and contributing significantly to functional impairment.

The review highlights the role of autoimmune mechanisms in driving neurological complications. Immune-mediated damage to the peripheral nervous system and CNS implicates autoantibodies such as anti-Ro/SSA and anti-La/SSB. Studies like Beh et al. [17] emphasized the contributions of immune dysregulation, inflammation, and vascular abnormalities to the pathogenesis of SS-related neurological symptoms. These autoantibodies were strongly associated with severe disease and a higher risk of neurological complications, highlighting the importance of immunological markers in clinical evaluations.

Autonomic dysfunction, another critical aspect of SS, was also highlighted. Scofield [18] reported that up to 50% of SS patients exhibit signs of autonomic impairment, including orthostatic hypotension, gastrointestinal disturbances, and dry eyes. These symptoms further complicate the clinical picture and necessitate careful management. Recognizing and addressing autonomic dysfunction is crucial for improving patient outcomes and enhancing the overall management of SS.

Accurate diagnosis of neurological complications in SS requires reliable and objective diagnostic tools. Neuroimaging techniques, such as brain and spinal MRIs and nerve conduction studies, were used in studies like Tumiati et al. [22] and Fox [23] to evaluate neurological involvement. These methods improved the accuracy of diagnoses, allowing for early detection and prompt intervention to better manage SS patients' neurological symptoms.

Treatment strategies for SS-related neurological complications remain challenging. Although no universally accepted therapies exist, immunotherapy, including corticosteroids and DMARDs, has shown promise in improving outcomes. Goodman [19] conducted an RCT demonstrating significant neurological improvements in SS patients receiving immunotherapy, particularly those with CNS symptoms. However, targeted therapies for conditions such as small-fiber neuropathy and cognitive dysfunction remain lacking. Studies like Davies and Ng [20] highlighted the critical need for treatments that address the underlying immune-mediated mechanisms driving these complications, as well as symptomatic management strategies such as pain control and cognitive rehabilitation.

Limitations of the study

Despite its comprehensive scope, this systematic review has several limitations. Most of the included studies were observational, limiting the ability to establish definitive causal relationships between SS and its neurological complications. Observational designs also introduce potential biases, such as selection and reporting biases, which may reduce the generalizability of the findings. Additionally, significant heterogeneity in study designs, diagnostic criteria, and outcome measures complicated direct comparisons of results. For example, Seeliger et al. employed different methods for assessing peripheral neuropathy, which may have influenced the reported prevalence rates [21].

Other limitations include some studies' relatively small sample sizes, which reduced statistical power and the ability to detect subtle trends or differences. The lack of long-term follow-up in many studies further restricted the understanding of the progression and chronicity of neurological complications in SS. Moreover, while some studies demonstrated promising therapeutic outcomes, the absence of RCTs and variability in treatment regimens prevented definitive conclusions about the most effective interventions. Finally, few studies accounted for the influence of comorbid conditions or genetic factors, which could play a significant role in the neurological manifestations of SS, further complicating the interpretation of findings.

Future directions

Future research on SS-related neurological complications must address several critical gaps identified in this review. Large-scale, multicenter, and longitudinal studies are necessary to explore the temporal progression of neurological symptoms and establish robust causal links between SS and its neurological manifestations. Ensuring diverse patient populations in these studies will also improve the applicability of findings across demographic groups. Additionally, standardization of diagnostic criteria and outcome measures is urgently needed to facilitate more reliable comparisons across studies and enhance clinical practice. Refining diagnostic tools for peripheral neuropathy, cognitive dysfunction, and autonomic dysfunction will enable early detection and intervention.

RCTs should be conducted to evaluate the efficacy of targeted therapies, particularly immunosuppressive and biological treatments, in mitigating neurological damage in SS patients. Research into novel therapeutic approaches, such as immune modulators, may offer additional options for addressing the underlying immune-mediated mechanisms driving these complications. Investigating the impact of comorbidities, such as cardiovascular disease, diabetes, and thyroid disorders, on neurological outcomes would provide valuable insights into the complex interactions influencing patient health. Finally, developing personalized treatment strategies based on individual genetic, clinical, and immunological profiles can revolutionize care for SS patients suffering from neurological complications.

## Conclusions

The findings underscore the imperative for early identification, accurate diagnostic methodologies, and efficacious therapeutic interventions to address these complications and improve the quality of life for patients with SS. While existing treatment modalities, including immunotherapy, demonstrate potential, this review highlights the pressing need to develop advanced, targeted therapies that effectively address the underlying immune-mediated mechanisms contributing to these neurological manifestations. By promoting sustained research efforts and fostering clinical advancements, this review conveys a sense of optimism for formulating improved management paradigms and enhanced outcomes for individuals afflicted by SS.
